# Tailorable Nanoparticles for Magnetic Water Cleaning of Polychlorinated Biphenyls

**DOI:** 10.1002/smtd.202500537

**Published:** 2025-05-08

**Authors:** Lukas Müller, Anna Zehetmeier, Anna Höfling, Henrik Gaß, Johannes Voß, Daniel Krappmann, Linda Rockmann, Elias Harrer, Dirk Zahn, Andreas Hirsch, Marcus Halik

**Affiliations:** ^1^ Organic Materials & Devices Institute of Polymer Materials Interdisciplinary Center for Nanostructured Films (IZNF) Friedrich‐Alexander‐Universität Erlangen‐Nürnberg 91058 Erlangen Germany; ^2^ Chair of Organic Chemistry II Friedrich‐Alexander‐Universität Erlangen‐Nürnberg 91058 Erlangen Germany; ^3^ Computer Chemistry Center Chair of Theoretical Chemistry Friedrich‐Alexander‐Universität Erlangen‐Nürnberg 91052 Erlangen Germany

**Keywords:** persistent organic pollutants, polychlorinated biphenyls, self‐assembled monolayers, superparamagnetic iron oxide nanoparticles, tailorable adsorption, water remediation

## Abstract

Anthropogenic persistent organic pollutants pose a pressing threat to the environment and human health. They can be found in water bodies all around the world at low but hazardous concentrations. Typical representatives of this contaminant class are polychlorinated biphenyls (PCBs). Here, nanoparticulate core‐shell water cleaning agents are presented, which are able to remove PCBs of various chlorination degrees from water. The core consists of superparamagnetic iron oxide nanoparticles (SPIONs) providing a large surface area that can be tuned via self‐assembled monolayers (SAMs) composed of phosphonic acid derivates. This shell binds the pollutants non‐covalently enabling facile magnetic water remediation. By employing positively charged or hydrophobic SAMs different PCBs can be preferentially removed. Furthermore, these orthogonal functionalities can be integrated into one SPION system. By combining charged and hydrophobic phosphonic acid derivates in so‐called binary SAMs the removal preference can be convoluted, which works just as well in real river water. The cost‐efficient availability of the base materials for these tailorable nanoparticles is complemented with recyclability laying the foundation for a sustainable water cleaning process.

## Graphical abstract

Polychlorinated biphenyls (PCBs) are human‐made persistent organic pollutants omnipresent in the earth's hydrosphere, which are reported hazardous to humans as well as wildlife. This study presents surface functionalized magnetic nanoparticles that can remove such contaminants from water with an adjustable preference for most concerning compounds.

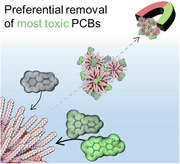

## 1 Introduction

According to the United Nations Environment Programme persistent organic pollutants (POPs) – despite being known for decades – are an increasing threat to humankind.^[^ ^1^ ^]^ They include anthropogenic molecules that, once entered, remain in the environment for a long time and get distributed around the world. Due to their hydrophobicity, they accumulate in living organisms while being toxic not only to wildlife but also to us humans.^[^ ^2^, ^3^ ^]^ For this reason the Stockholm Convention on Persistent Organic Pollutants or short just Stockholm Convention hosted by the United Nations was put into action in 2004 as an international measure against POPs.^[^ ^4^ ^]^ Among the twelve initial pollutants of concern are the so‐called polychlorinated biphenyls (PCBs). Consisting of two phenyl rings, which can be chlorinated at up to ten positions, they form a group of 209 congeners typically occurring in mixtures. Due to their chemical stability, temperature resistance, and dielectric properties, they have been widely used, for example, in closed electrical equipment but also openly as plasticizers in polymers and paints.^[^ ^5^, ^6^ ^]^ It was estimated that between 1 and 1.5 million tons of PCBs were produced worldwide since the 1920s until being mostly banned in the 1980s and 90s.^[^ ^7^ ^]^ Because of improper discharge and high environmental mobility, they are not only found around urban industrialized places like Munich in Germany,^[^ ^8^ ^]^ the San Francisco Bay in the United States^[^ ^9^ ^]^ or megalopolises in China^[^ ^10^ ^]^ but also in more rural areas like the Himalayan riverine network in Pakistan^[^ ^11^ ^]^ or down at the Mariana trench in the Pacific Ocean.^[^ ^12^ ^]^ The omnipresence is particularly concerning as it is known that PCBs negatively influence early childhood development^[^ ^13^, ^14^ ^]^ and are declared carcinogenic^[^ ^15^ ^]^ among other harmful effects.^[^ ^16^ ^]^ In the river Nile in Egypt concentrations of PCBs between 14 and 20 µg L^−1^ were measured,^[^ ^17^ ^]^ while the United States Environmental Protection Agency enforces a maximum contaminant level of 0.5 µg L^−1^ with the declared goal of zero residues in their national primary drinking water regulations.^[^ ^18^ ^]^ In fact, the Stockholm Convention set the sophisticated aim of complete and environmentally friendly elimination of PCBs until 2028 to obey the sustainable development goals (SDGs) of the United Nations.^[^ ^19^ ^]^ Therefore, it is necessary to efficiently clean wastewater from PCBs at very low concentrations, so‐called trace concentrations. In Germany, more than 96 % of wastewater is treated by conventional wastewater treatment plants (WWTPs), which typically consist of a mechanical, a biological, and a precipitation stage.^[^ ^20^ ^]^ However, even state‐of‐the‐art WWTPs having an additional fourth stage comprised of oxidation via ozonation and/or activated carbon filters cannot remove all trace contaminants sufficiently.^[^ ^21^ ^]^ Consequently, advanced water cleaning is an active field of research. One way to classify approaches is by their biological, chemical, and physical (or combined) nature.^[^ ^22^, ^23^, ^24^ ^]^ Microbial remediation of PCBs, i.e., degradation by microorganisms like *A. xylosoxidans*,^[^ ^25^ ^]^ is a low energy‐consuming and environmentally‐friendly approach. However, it takes a long time and works less effectively for stable chlorination at ortho‐positions on the phenyl rings.^[^ ^24^ ^]^ Therefore, oxidation processes like the aforementioned ozonation or treatment with Fenton's reagent (Fe^2+^/H_2_O_2_),^[^ ^26^ ^]^ photocatalytic degradation^[^ ^27^ ^]^ as well as dechlorination by reduction as shown with zero‐valent iron^[^ ^28^ ^]^ provide more versatile degradation strength. Here, the cost and environmental impact of the treatment itself are the issues of concern.^[^ ^23^ ^]^ Next to membrane filtration, which is inherently limited for large water volumes, POPs‐attractive adsorbents providing large surface areas resemble a simple and cost‐effective concept.^[^ ^29^ ^]^ As mentioned before, activated carbon is one of the most studied and already employed adsorbents for water remediation today providing high surface area but type‐dependent PCB adsorption efficiencies.^[^ ^21^, ^30^, ^31^ ^]^ Therefore, costly modification processes are necessary to target specific pollutants.^[^ ^32^ ^]^ Another elegant approach is to use superparamagnetic iron oxide nanoparticles (SPIONs), known for their theranostic medical applications^[^ ^33^ ^]^ or as precursors for Fischer‐Tropsch catalysts,^[^ ^34^, ^35^ ^]^ as adsorbents to enable removal from the water phase by applying a magnetic field gradient.^[^ ^29^ ^]^ Examples show SPIONs incorporated in highly porous metal‐organic frameworks^[^ ^36^ ^]^ as well as SPIONs coated with complex molecules like β‐cyclodextrins^[^ ^37^ ^]^ or tediously synthesized polymeric shells^[^ ^38^ ^]^ to make them attractive for PCBs.

**Figure 1:**
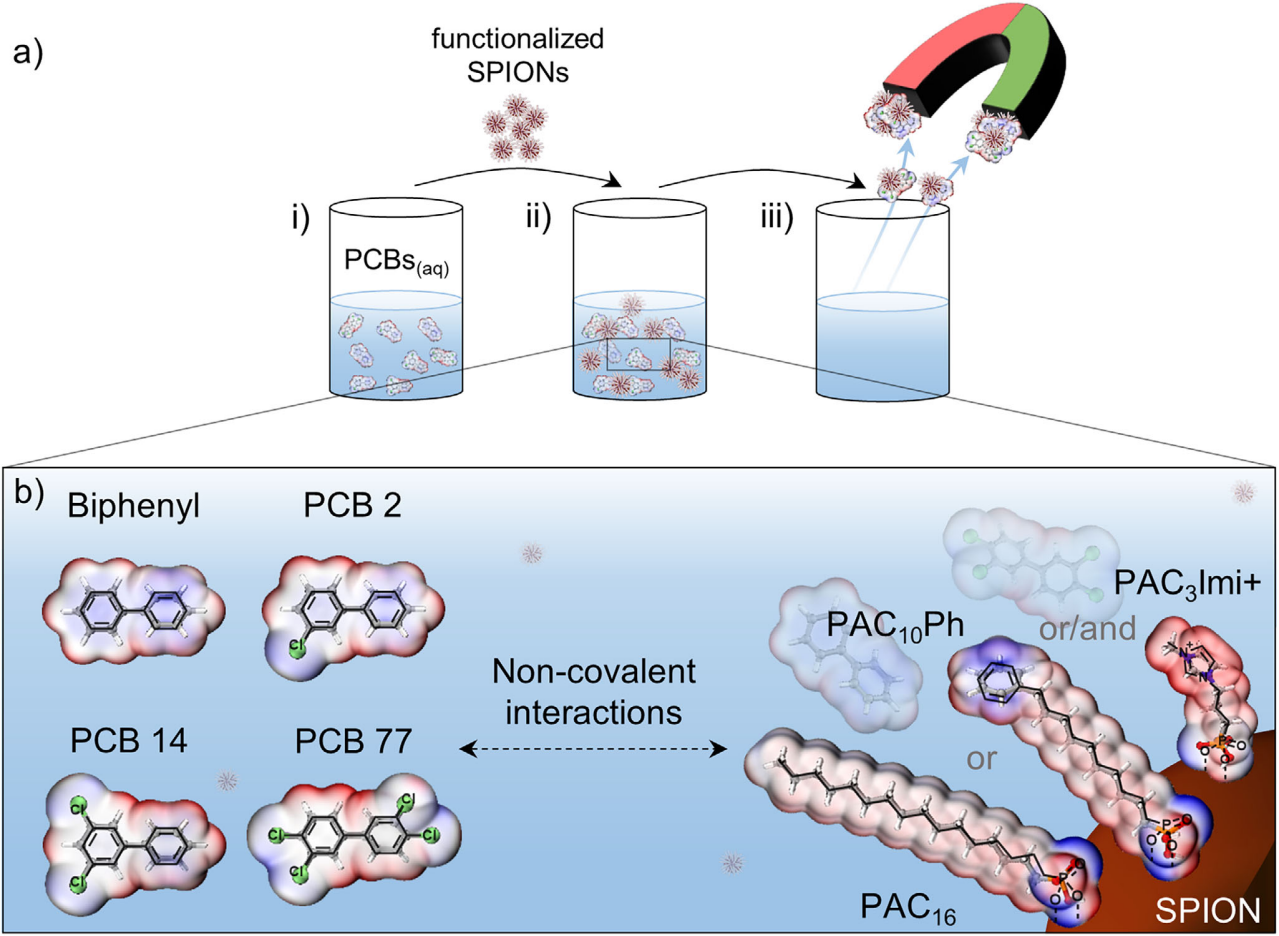
Schematic approach of magnetically removing PCBs from water via surface‐functionalized SPIONs. a) An aqueous PCB solution i) is treated with functionalized SPIONs, onto which the contaminants adsorb ii). The SPIONs can be magnetically collected carrying the PCBs iii). b) Scope of investigated PCBs (Biphenyl, PCB 2, PCB 14, and PCB 77) as well as SPION surface modifications (PAC_16_, PAC_10_Ph, PAC_3_Imi+, PAC_3_Imi+/PAC_16,_ and PAC_3_Imi+/PAC_10_Ph) tailored to address the PCBs non‐covalently. The electrostatic potential maps were calculated for electron density isovalue surfaces as described in the supporting information and range from −0.8 V (blue) to +0.8 V (red) for all molecules except PAC_3_Imi+ where red indicates +5.3 V.

Here, we present ton‐scale produced SPIONs coated with PCB‐attractive self‐assembled monolayers (SAMs) consisting of simple, commercially available, and strongly binding phosphonic acid derivates (PA_i_@SPIONs). Comparable core‐shell SPION systems have previously been shown to be not only non‐toxic^[^ ^39^, ^40^ ^]^ but also very efficient for removing a broad range of simple hydrocarbons,^[^ ^41^, ^42^ ^]^ charged dyes^[^ ^43^ ^]^ as well as micro‐ and nanoplastics of various sizes from water.^[^ ^40^, ^44^ ^]^ As **Figure** **1**a illustrates, via molecular self‐assembly functionalized nanoparticles can be added to aqueous PCB solutions so that the large functional surface can interact non‐covalently with the pollutants. The superparamagnetism of the cores enables facile magnetic PCB extraction from the aqueous solution, which is quantified by washing the contaminant‐carrying SPIONs in hexane and analyzing the PCB concentration via gas chromatography coupled to mass spectrometry (GC‐MS). As representatives for the 209 congeners, non‐chlorinated 1,1′‐biphenyl (biphenyl), once‐chlorinated 3‐chloro‐1,1′‐biphenyl (PCB 2), twice‐chlorinated 3,5‐dichloro‐1,1′‐biphenyl (PCB 14) and four times‐chlorinated 3,3′,4,4′‐tetrachloro‐1,1′‐biphenyl (PCB 77) are removed individually but also competitively in mixtures in dependence of the functional surface of the SPIONs (see Figure 1b).

The SPION surface is complementarily decorated with three different phosphonic acid derivates to address the PCBs. 1‐Methyl‐3‐(propylphosphonic acid) imidazolium bromide (simplified as the cation only PAC_3_Imi+) is used to provide positive charges on the SPION surface for attractive electrostatic interactions with the electronegative chlorine atoms. On the other hand, n‐hexadecylphosphonic acid (PAC_16_) and 10‐phenyldecylphosphonic acid (PAC_10_Ph) provide hydrophobic interaction sites combined with, in the latter case, an additional electron‐rich phenyl ring to potentially interact with the electron‐deficient rings of the chlorinated pollutants. The electronic properties of the molecules are featured in Figure 1b by means of electrostatic potential maps, which were calculated by density functional theory (see Supporting Information). In suitable contaminant‐to‐SPION ratios that enable quantitative comparison between the systems, the preferred interaction of biphenyl, PCB 2, and PCB 14 with hydrophobic PAC_16_@SPIONs and PAC_10_Ph@SPIONs is shown, while PCB 77 – despite being less water soluble than the other pollutants^[^ ^45^ ^]^ – is more efficiently extracted by the positively charged PAC_3_Imi+@SPIONs. Moreover, these orthogonal surface functionalities can be combined on the SPION surface by a mixed (binary) SAM^[^ ^46^, ^47^, ^48^ ^]^ (PA_i_/PA_j_@SPIONs) comprising PAC_3_Imi+ and PAC_16_ or PAC_10_Ph translating into tailorable adsorption behavior between the extrema. These nanoparticulate water cleaning agents do not only remove the investigated PCBs just as well from river water (Regnitz in Erlangen, Germany) but can be recycled without any sign of degradation and only small performance losses for three out of four pollutants. Altogether, the simplicity of the magnetic cleaning process, cost‐efficient availability of the base components as well as versatility in approaching different environmental pollutants by variation of the surface chemistry suggest phosphonic acid functionalized SPIONs as a promising platform material for large‐scale water remediation. As demonstrated here, the incorporation of multiple non‐covalent interaction motifs in one SPION system underlines this potential as it enables both broadband as well as targeted water cleaning.

## 2 Results and Discussion

### 2.1 SPION Characterization

In this study, SPIONs (primary diameter of ≈10.7 nm providing a large specific surface area of 115.1 m^2^ g^−1^) were decorated with three different phosphonic acid derivates. PAC_3_Imi+, PAC_16_, and PAC_10_Ph bind to the nanoparticle surface covalently forming SAMs in the process as shown in Figure S1 and S2 (see Supporting Information) via attenuated total reflectance Fourier transform infrared spectroscopy (ATR‐FTIR) and thermogravimetric analysis. PAC_3_Imi+ renders the SPION surface more positively charged in comparison to uncoated particles as indicated by an increase in ζ‐potential at pH 7, while the other two molecules reduce it (see Figure S3, Supporting Information). However, these positive charges repel each other leading to a lower grafting density on the surface of 1.21 ± 0.08 nm^−2^ compared to the hydrophobic‐rendering PAC_16_ (2.25 ± 0.06 nm^−2^) and PAC_10_Ph (2.17 ± 0.11 nm^−2^). This less dense packing is exploited by a second self‐assembly step with the alkyl molecules in order to form binary SAMs on the nanoparticle surface. These systems, PAC_3_Imi+/PAC_16_@SPIONs, and PAC_3_Imi+/PAC_10_Ph@SPIONs, combine the orthogonal properties of the individually modified SPIONs (increasing vs decreasing ζ‐potential, less vs more dense) on one surface (see Figures S1–S3, Supporting Information).

### 2.2 Magnetic Biphenyl and PCB Extraction

All magnetic extractions were performed at neutral pH with a fixed pollutant‐SPIONs ratio (7 mL à 1 µm of PCB, i.e., 7 nmol were treated with 5.0 ± 0.1 mg of SPIONs). This ratio was deliberately chosen to enable quantitative comparison between the systems. Such low concentrations require robust analytical quantification. Based on recommendations by the United States Environmental Protection Agency on quantification of PCBs,^[^ ^49^ ^]^ this was achieved by washing the adsorbent systems after magnetic water cleaning with hexane to resolubilize all non‐covalently collected pollutants followed by analysis via GC‐MS. To each sample, an internal standard (decachloro‐1,1′‐biphenyl, PCB 209) at a fixed concentration was added. Representative chromatograms and calibration series of varying analyte to standard concentrations are given in Figure S4 (see Supporting Information).

**Figure 2:**
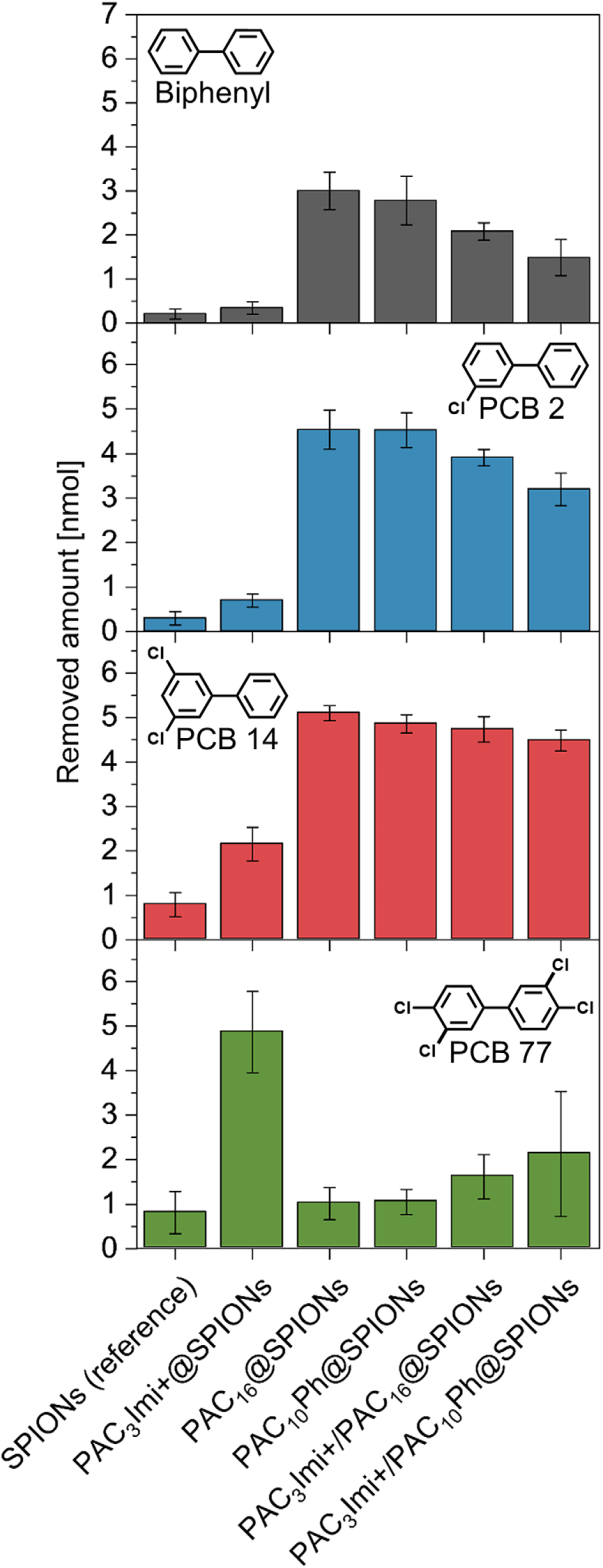
Magnetically removed amounts of biphenyl and PCBs depending on the molecular coating of the SPIONs. Aqueous solutions of 7 mL initially contained 7 nmol (1 µm) of pollutant at neutral pH and were treated with 5.0 ± 0.1 mg of SPIONs. Hydrophobic PAC_16_@SPIONs and PAC_10_Ph@SPIONs remove biphenyl, PCB 2, and PCB 14 more efficiently with a trending increase for less water solubility, while PAC_3_Imi+@SPIONs drastically outperforms them for PCB 77 despite it being the least water‐soluble. Binary SAMs on SPIONs show performances in between the extrema. Data are represented as mean ± standard deviation (*n* = 6).

The two hydrophobically‐rendered systems, PAC_16_@SPIONs, and PAC_10_Ph@SPIONs, the positively charged PAC_3_Imi+@SPIONs as well as their orthogonal binary SAM adsorbent systems (all in reference to uncoated SPIONs) were applied to aqueous biphenyl and PCB solutions. The magnetically extracted biphenyl and PCB amounts at the fixed pollutant‐SPION ratio are summarized in **Figure** **2**. First, the molecular SAMs enable efficient magnetic collection overall. The uncoated SPIONs (reference) peak at 0.80 ± 0.47 nmol removal for PCB 77 while only extracting 0.20 ± 0.11 nmol for biphenyl. On the other hand, PAC_16_@SPIONs is able to remove 3.00 ± 0.42 nmol of biphenyl and up to 5.10 ± 0.17 nmol for PCB 14 with a strong drop in performance for PCB 77. While PAC_10_Ph@SPIONs behaves similarly, PAC_3_Imi+@SPIONs shows as low removal as uncoated SPIONs for biphenyl, however, the highest magnetic extraction performance at all for PCB 77 of 4.86 ± 0.91 nmol. PAC_3_Imi+/PAC_16_@SPIONs has the same trend as PAC_16_@SPIONs while extracting biphenyl, PCB 2 and PCB 14 less and PCB 77 better. Furthermore, PAC_3_Imi+/PAC_10_Ph@SPIONs follows this trend even more pronounced.

These results indicate an interplay of hydrophobic effect‐driven segregation (i.e., hydrophobic segregation) and electrostatic interaction as the two major non‐covalent interaction motifs between the investigated pollutants and the functionalized adsorbent surface.^[^ ^50^ ^]^ PCBs are less water soluble with increasing chlorination degree.^[^ ^45^ ^]^ For biphenyl, PCB 2, and PCB 14 a respective increase in removal amounts is seen for all adsorbent systems corresponding to their decreasing water solubility. This in combination with the performance of PAC_16_@SPIONs and PAC_10_Ph@SPIONs supports a strong hydrophobic segregation on the nanoparticle surface for these pollutants. These adsorbents, however, lose a lot of performance for PCB 77 despite it being the least water‐soluble. Here, PAC_3_Imi+@SPIONs works best. This may be due to the positive charges on the surface that can attract electron‐rich chlorine atoms, of which PCB 77 comprises the most among the investigated molecules.

For the binary SAM‐coated SPIONs, the characterized combination of orthogonal properties of charged PAC_3_Imi+@SPIONs and hydrophobic PAC_16_@SPIONs or PAC_10_Ph@SPIONs translates into their interaction with the pollutant molecules. The extraction is dominated by the properties of the alkyl part of the SAM, however, complemented with the influence of PAC_3_Imi+ (compare ζ‐potentials in Figure S3, Supporting Information) enabling average removal amounts in between the unitary SAMs. As mentioned before, PAC_10_Ph was used not only to serve hydrophobic interaction sites but also to provide electron‐rich phenyl rings for potential interaction with electron‐deficient, chlorinated phenyl rings of the PCBs. While the magnetic extraction performance of PAC_16_@SPIONs and PAC_10_Ph@SPIONs differs only insignificantly, PAC_3_Imi+/PAC_10_Ph@SPIONs shows on average a worse performance for biphenyl and PCB 2, a comparable one for PCB 14 and an increase for PCB 77 (with large variation in between multiplicates) in reference to the other binary coated system. This points to a potential second, weaker instance of electrostatic interaction: repulsion between electron‐rich rings of biphenyl and PCB 2 and the electron‐rich phenyl ring in PAC_10_Ph as well as attraction of electron‐deficient rings in PCB 77 toward these.

### 2.3 Magnetic Extraction of Biphenyl and PCB Mixtures

**Figure 3:**
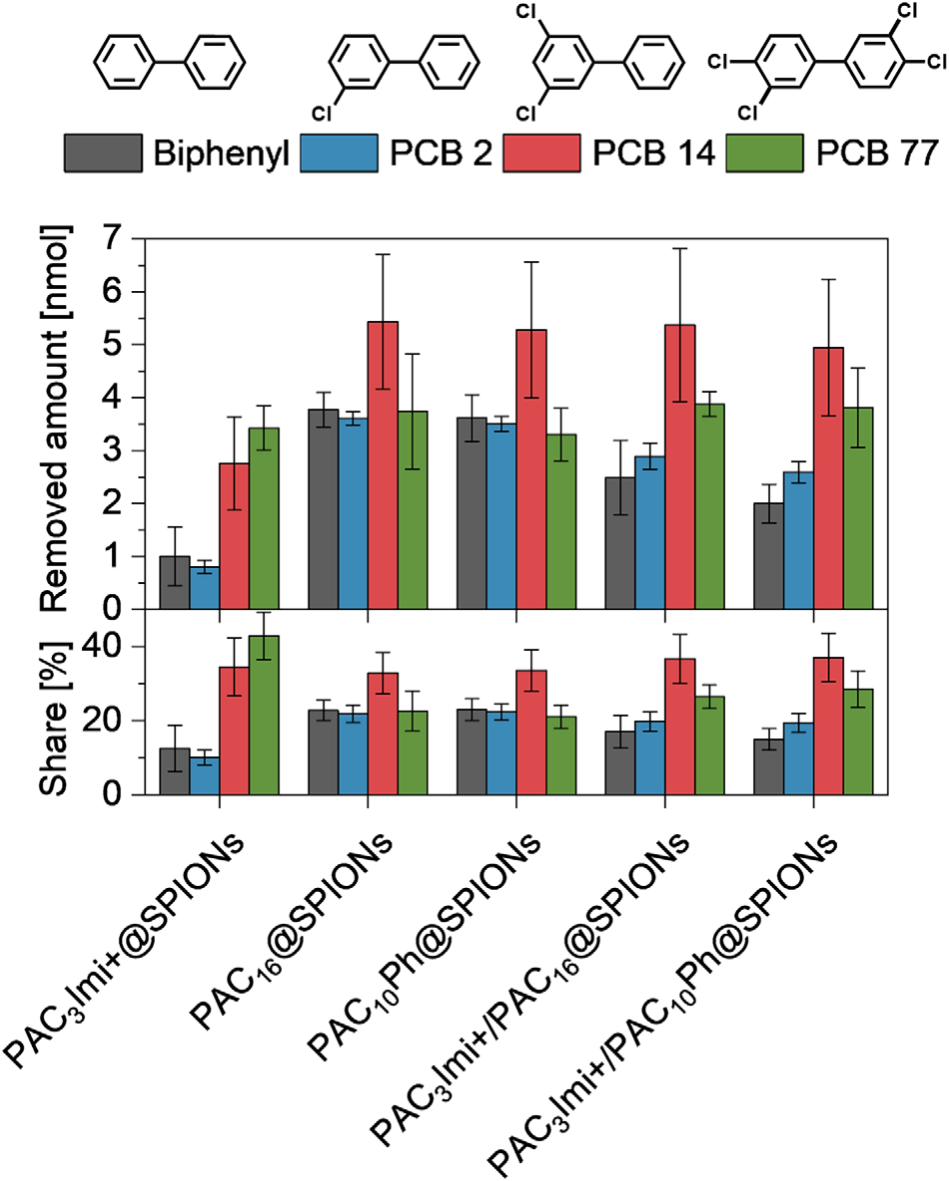
Magnetically removed amounts of biphenyl and PCBs from mixtures depending on the molecular coating of the SPIONs. Aqueous solutions of 7 mL initially contained 7 nmol (1 µm) of each pollutant, i.e., 28 nmol in sum, at neutral pH and were treated with 5.0 ± 0.1 mg of SPIONs. The top graph shows magnetically removed amounts and the bottom graph the respective normed shares of biphenyl and each PCB. Overall, PCB 14 is removed best by the hydrophobic adsorbents. The preference of PAC_3_Imi+@SPIONs for PCB 77 can be integrated into the behavior of the hydrophobic systems as results with PAC_3_Imi+/PAC_16_@SPIONs and PAC_3_Imi+/PAC_10_Ph@SPIONs show. Data are represented as mean ± (Gaussian error propagated) standard deviation (*n* = 6).

In the environment PCBs usually occur in mixtures.^[^ ^5^ ^]^ Therefore, also mixtures of the investigated biphenyl and PCBs were magnetically treated with SPION systems (now containing 1 µm of each pollutant, i.e., 28 nmol overall) as presented in **Figure** **3**. In addition to the removed amounts (top), the normed shares of each pollutant in the removed amount are given (bottom) enabling better pollutant interaction preference discussion between the systems. Overall, the combined magnetically removed amounts are increased for the treatment of mixtures. PAC_3_Imi+@SPIONs extracts a sum of 7.98 ± 1.13 nmol of pollutants with the highest share of 42.9% ± 6.4% coming from PCB 77, 34.5% ± 7.8% from PCB 14, 12.5% ± 6.3% from biphenyl and 10.0% ± 2.0% from PCB 2. Again, PAC_16_@SPIONs and PAC_10_Ph@SPIONs behave similarly by extracting 16.55 ± 1.71 and 15.69 ± 1.45 nmol, respectively, with the highest share stemming from PCB 14 (≈33% in both cases) and no significant deviation between the shares of biphenyl, PCB 2 and PCB 77 (≈21%–23% each). In general, multiplicates show relatively large variations for PCB 14. With the binary coated PAC_3_Imi+/PAC_16_@SPIONs and PAC_3_Imi+/PAC_10_Ph@SPIONs overall 14.62 ± 1.65 and 13.34 ± 1.55 nmol of pollutants are removed. Here, PCB 14 also makes the biggest share of ≈37% in both cases, while the share of PCB 77 is estimated to 26.5% ± 3.2% and 28.5% ± 5.0%, respectively. The shares of biphenyl removed by PAC_3_Imi+/PAC_16_@SPIONs and PAC_3_Imi+/PAC_10_Ph@SPIONs are 17.0% ± 4.4% and 15.0% ± 2.9% and, therefore, being lower than ≈20 % for PCB 2.

At the previously introduced maximum contaminant level of 0.5 µg L^−1^ set by the United States Environmental Protection Agency every liter contains ≈2.3 nmol of PCBs calculated with the average molar mass of the here investigated pollutants. The SPIONs could be able to adsorb this amount in a single treatment. The performances of the different adsorbent systems for the treatment of mixtures of biphenyl and PCBs mostly resemble the trends of the separate extractions. The overall higher removal amounts need to be considered under the influence of higher starting amounts. Hydrophobic segregation leads to PAC_16_@SPIONs and PAC_10_Ph@SPIONs extracting the highest amounts of pollutants with the biggest share of PCB 14. PCB 77 is not significantly worse extracted than biphenyl and PCB 2 by these systems in contrast to the separate treatments indicating potential interaction among the pollutants. Adsorbed electron‐rich phenyl rings, e.g., of biphenyl, may interact with the deficient rings of PCB 77 leading to better magnetic extraction. PAC_3_Imi+@SPIONs extracted the smallest sum of contaminant molecules overall but showed a higher preference for PCB 77 despite it being the least water‐soluble as mentioned before, which should lead to the highest preference for adsorption onto hydrophobic surfaces instead. This confirms the previously identified electrostatic interaction as the second major interaction force at play. The electronegative chlorine atoms are attracted by the imidazolium cations. The binary coated systems, PAC_3_Imi+/PAC_16_@SPIONs, and PAC_3_Imi+/PAC_10_Ph@SPIONs, again integrate both results in one system. The overall higher extraction amounts with peak in PCB 14 observed for the hydrophobic adsorbents are maintained while incorporating the higher affinity for PCB 77 in comparison to biphenyl and PCB 2 coming from the electrostatic interaction sites provided by PAC_3_Imi+. Here, the electron‐rich phenyl ring in PAC_10_Ph provides no further significant interaction motif. The preference for removing PCB 77 is of particular interest as it is coplanar, substituted in both para and two meta positions while being non‐ortho‐substituted representing the most toxic PCBs.^[^ ^51^ ^]^

### 2.4 Approaching More Realistic Cleaning Conditions and Constraints

**Figure 4:**
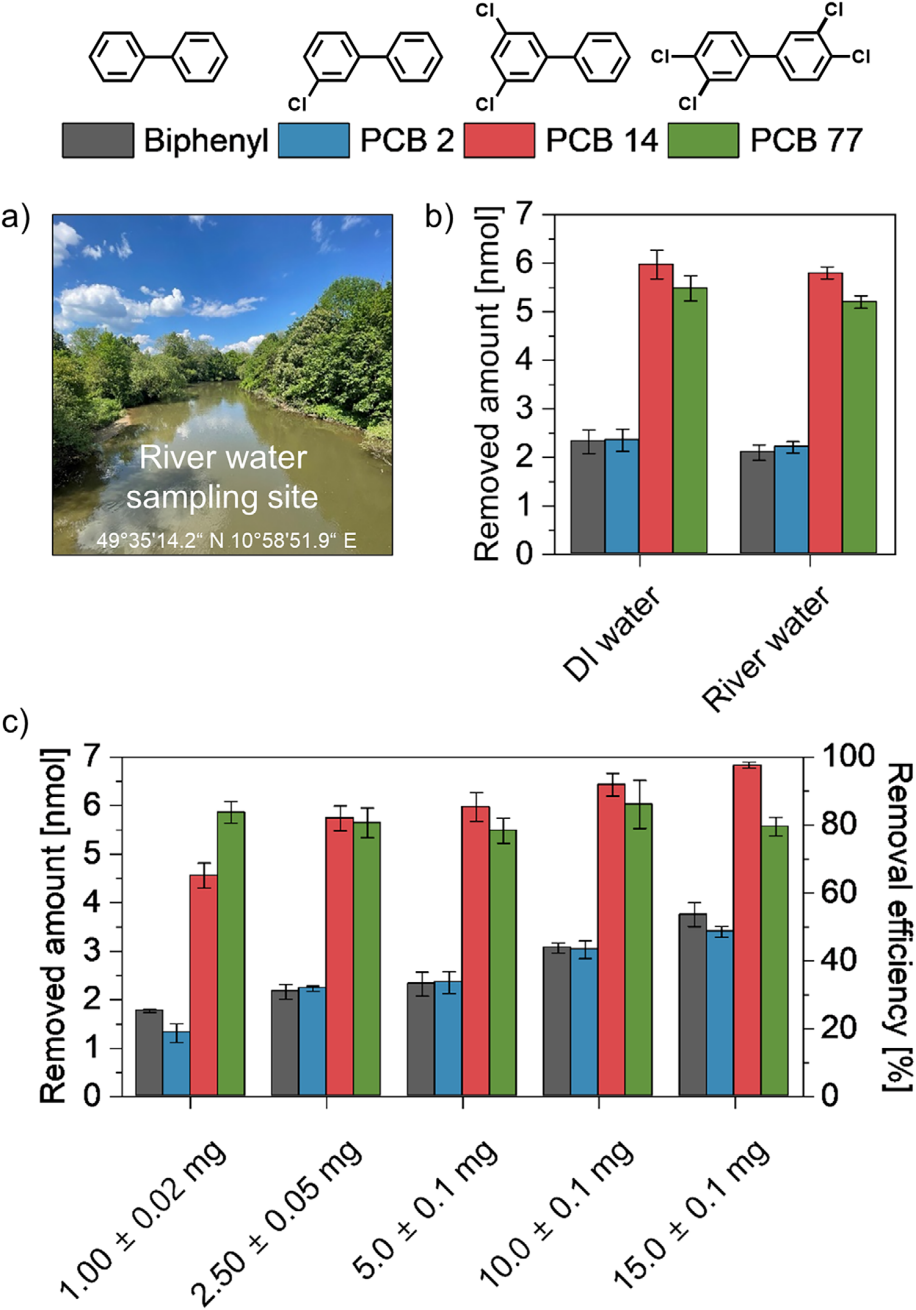
Magnetically removed amounts of biphenyl and PCBs from mixtures in DI versus river water as well as efficiency dependence on the employed mass on the example of PAC_3_Imi+/PAC_10_Ph@SPIONs. a) Photograph of river water sampling site, in which the decorated SPIONs work as well as in DI water as seen in b). Aqueous solutions of 7 mL initially contained 7 nmol (1 µm) of each pollutant, i.e., 28 nmol in sum. In DI water the pH was neutral, while the natural value of 7.95 was maintained in river water. The employed mass of PAC_3_Imi+/PAC_10_Ph@SPIONs in b) was 5.0 ± 0.1 mg. c) Variation of the employed mass of these SPIONs for treatment of DI water spiked with equally concentrated PCB mixtures as in b) shows that more SPIONs yield higher removal efficiencies of up to 97.6 % ± 0.9 % as demonstrated for PCB 14. Data are represented as mean ± standard deviation (*n* = 3).

In realistic applications mixtures of biphenyl and PCBs would not be present in a matrix of deionized (DI) water, but in much more complex, natural water matrices. As investigated before on other examples,^[^ ^40^, ^52^ ^]^ the performance of the presented adsorbent systems, represented by PAC_3_Imi+/PAC_10_Ph@SPIONs, was tested on biphenyl and PCB mixtures in actual river water collected from the Regnitz in Erlangen, Germany (49°35′14.2″ N 10°58′51.9″ E) as illustrated in **Figure** **4**a,b. Removing ≈15–16 nmol of pollutants both from DI and river water matrices with an adsorption share of ≈37 %–38 % for PCB 14 and ≈34 % for the most toxic PCB 77, the PAC_3_Imi+/PAC_10_Ph@SPIONs work just as well in this more realistic scenario. The differences in comparison to Figure 3 are likely due to strong influences of minor concentration fluctuations in the starting solutions. The data in Figure 3 was averaged across twice as many multiplicates. So far, all extractions were performed with the same employed mass of SPIONs to identify the role of the different functional coatings. In Figure 4c the employed mass is varied for constant starting amounts of 7 nmol each of biphenyl and PCBs in mixtures. Next to the removed amounts, this time also the efficiencies are given. While with the previously tested mass of 5.0 ± 0.1 mg of PAC_3_Imi+/PAC_10_Ph@SPIONs ≈16 nmol of PCBs are removed, decreasing the mass by a factor of five to 1.00 ± 0.02 mg still removes ≈13 nmol overall. By increasing the mass to 15.0 ± 0.1 mg ≈20 nmol are removed. Overall, the removal efficiencies of biphenyl, PCB 2, and PCB 14 can be increased by employing more SPIONs with a peak for PCB 14 of 97.6 % ± 0.9 % removal using 15.0 ± 0.1 mg of PAC_3_Imi+/PAC_10_Ph@SPIONs. In contrast, the efficiency for PCB 77 remains constant for all employed masses at ≈80 %. It should be mentioned that the removal efficiency is found to be non‐linearly dependent on the employed mass and it appears that already the smallest mass of employed SPIONs apparently provides an excess of adsorption surface, which concludes a non‐saturated adsorbent at the investigated setup and, thus, makes extrapolation hardly possible. The efficiency values should be regarded as proof‐of‐concept only.

Another aspect to be considered is the recyclability of the adsorbent. Again, with the same surrogate SPION system repeated water treatments of biphenyl and PCB mixtures were conducted. As ATR‐FTIR spectra and thermogravimetric analyses show, no degradation of the binary SAM on the SPIONs can be observed after three cycles, nor do any detectable amounts of pollutants remain on the particle surface after hexane washing (see Figure S5, Supporting Information). In terms of removal performance, the removed amount of 15.34 ± 0.82 nmol in the first cycle drops by ≈38 % for the second and another 12 % for the third cycle mostly by a decrease in the removed amount of PCB 77 (see Figure S6, Supporting Information). This could be due to remaining traces of hexane in the shell from the washing process below the detection limit that potentially block imidazolium moieties and compete with the adsorption of PCB 77. The adsorption of hexane is intuitive from previous work on small hydrocarbons, which bind on similar SAMs.^[^ ^41^, ^42^ ^]^ Nevertheless, the surrogate SPIONs are found to be recyclable.

## 3 Conclusion

In this study, we presented nanoparticulate water cleaning agents based on ton‐scale produced SPIONs covered with SAMs composed of commercially available phosphonic acid derivates. These simple and non‐toxic^[^ ^39^, ^40^ ^]^ adsorbents enable magnetic extraction of POP‐representative biphenyl, mono‐chlorinated PCB 2, di‐chlorinated PCB 14, and tetra‐chlorinated PCB 77 from aqueous solutions at low concentrations. An interplay of hydrophobic segregation and electrostatic interactions causes the non‐covalent attraction of the pollutants to the functionalized nanoparticle surface. The nature of this surface is tailorable by choice of the phosphonic acid derivate SAM. PAC_3_Imi+@SPIONs provides positively charged interaction sites, while PAC_16_@SPIONs and PAC_10_Ph@SPIONs equip the adsorbent surface with a hydrophobic interface, which translates into corresponding interactions with the pollutants. Since the water solubility of PCBs decreases with increasing degree of chlorination,^[^ ^45^ ^]^ increasing segregation from biphenyl to PCB 14 onto PAC_16_@SPIONs and PAC_10_Ph@SPIONs is observed. Counterintuitively, this inverts for the least water‐soluble PCB 77, which is removed best by PAC_3_Imi+@SPIONs. This is attributed to electrostatic interactions between the cationic imidazolium and electronegative chlorine atoms. The orthogonal properties of these SAMs can be integrated into single adsorbent systems with binary SAMs comprising PAC_3_Imi+ and PAC_16_ or PAC_10_Ph on one surface. These systems show magnetic extraction performances in between the extrema while being dominated by the influence of the uncharged molecules. Since PCBs usually occur in mixtures, the pollutants were also removed in competition. Here, the described trends continue with PAC_3_Imi+@SPIONs showing the highest affinity to PCB 77, while the hydrophobic adsorbents extract PCB 14 best. A convolution of both behaviors is found for the binary SAM‐coated systems providing the highest removal amounts for PCB 14 while having a preference for PCB 77 over biphenyl and PCB 2, which is of particular interest as PCB 77 is among the most toxic PCBs.^[^ ^51^ ^]^ With PAC_3_Imi+/PAC_10_Ph@SPIONs as a surrogate, it was shown that magnetic extraction of PCB mixtures works just as well in a natural water matrix, i.e., river water collected from the Regnitz in Erlangen, Germany. By employing more SPIONs higher removal efficiencies can be achieved approaching up to 97.6 % ± 0.9 % removal in a single treatment as demonstrated for PCB 14. Furthermore, the recyclability of these adsorbents was shown. The binary SAM does not degrade over three cycles of application. Ultimately, we believe that the demonstrated approach of magnetic water cleaning of PCBs is very promising as cost‐efficiently removing POPs at low concentrations from water remains a challenge. Being able to address individual components of pollutant molecule mixtures preferentially by tailoring the molecular shell on large surface area providing nanoadsorbents may be beneficial in the future considering the majority of pollutants is still out there.^[^ ^19^ ^]^

## 4 Experimental Section

### Materials

SPIONs were provided by Comar Chemicals (Pty). These particles show the crystal structure of maghemite (γ‐Fe_2_O_3_) as determined previously^[^ ^40^ ^]^ and have an average primary diameter of 10.7 nm assuming maghemite bulk density and spherical shape calculated from their Brunauer‐Emmett‐Teller (BET) specific surface area (*SSA_SPIONs_*) of 115.1 m^2^ g^−1^ measured via N_2_ gas adsorption with a Nova 4000e from Quantachrome. More details can be found in the Supporting Information. Phosphonic acid derivates PAC_3_Imi+ (≥95%) and PAC_10_Ph (≥95%) were purchased from SiKÉMIA, while PAC_16_ was obtained from PCI Synthesis. The contaminants biphenyl (99.5 %), PCB 2 (analytical standard), PCB 14 (analytical standard) as well as the quantification internal standard PCB 209 (analytical standard) stem from Sigma‐Aldrich. PCB 77 (99.8 %) was purchased from LGC Standards. As solvents methanol (≥99%), acetone (≥99.5%), and hexane (≥99%) from Carl Roth as well as DI water from the DI tap of the Interdisciplinary Center for Nanostructured Films building were used. The river water was taken from the Regnitz in Erlangen, Germany (49°35′14.2“ N 10°58′51.9” E) on April 3, 2024. After removing coarse sediments by decanting, it was filtered twice through a nylon stocking (30 DEN, 33 dtex) and stored in a brown glass bottle.

### SPION Functionalization

Methanol solutions à 5 mm for PAC_3_Imi+ or 10 mm for PAC_16_ and PAC_10_Ph were added to dispersions of SPIONs (3 mg mL^−1^) also in methanol at a volumetric ratio of 3:10, respectively. These concentrations were determined to saturate the surface beforehand. The mixture was sonicated for 30 min at 25 °C in a Sonocool 255 from Bandelin. Unbound phosphonic acid molecules were removed by two washing cycles consisting of sedimenting the functionalized SPIONs with a Multifuge X1R from Heraeus (13000 rpm at 5 °C) followed by redispersion in fresh methanol. Finally, the coated SPIONs were sedimented again and dried at 60 °C overnight. For binary shells, the PAC_3_Imi+@SPIONs were not dried in the last step but redispersed in the initial methanol volume and then mixed with solutions of PAC_16_ or PAC_10_Ph à 10 mm in the same ratio as for the first iteration. Sonication as well as both washing cycles were repeated and the particles finally dried at 60 °C overnight.

### SPION Characterization

The functionalization of the SPIONs was qualitatively confirmed by ATR‐FTIR recorded with an IR Prestige 21 from Shimadzu between 650 and 4000 cm^−1^ at a resolution of 4 cm^−1^. The data was corrected with the internal atmospheric correction (Shimadzu IRsolution 1.60) removing residual H_2_O and CO_2_ vibration bands and normalized to the interval borders 650 and 4000 cm^−1^. Quantitatively, the particles were thermogravimetrically analyzed with a TG 209 F1 Libra from Netzsch. The temperature was continuously increased to 1000 °C at 30 °C min^−1^ while being exposed to a gas flow of 10 mL min^−1^ of O_2_ and 40 mL min^−1^ of N_2_. The measured curves were smoothed with the software Netzsch Proteus Thermal Analysis 8.0.3. The grafting densities of the phosphonic acids (*GD_PA_*) on the SPIONs’ surface were estimated via Equation (1). *wt* is the relative mass loss between 150 and 700 °C in percent, *N_A_* is Avogadro's constant, *MW_PA_* is the molar mass of the respective phosphonic acid derivate (or of PAC_16_/PAC_10_Ph respectively for the binary coated SPIONs as they make up the majority (on average >90%) of the coating as determined by ATR‐FTIR) corrected by the staying anchor group^[^ ^53^ ^]^ and *SSA_SPIONs_* the specific surface area of the SPIONs.

(1) $$GD_{\mathrm{PA}}=\frac{\mathrm{wt}}{100-\mathrm{wt}}\;\cdot \frac{N_{A}}{MW_{\mathrm{PA}}\cdot \mathrm{SS}A_{\mathrm{SPIO}\mathrm{Ns}}}$$

Hydrodynamic diameters (given as Z‐averages) and ζ‐potentials of particles in dispersion were determined via dynamic and electrophoretic light scattering, respectively, with a Zetasizer Nano ZS running software version 7.13 from Malvern. Here, the SPIONs were not dried after functionalization but redispersed in DI water. A reservoir of DI water was titrated to the desired pH with aqueous 0.25 m NaOH and HCl solutions and spiked with the SPION dispersion. The obtained pH value was noted afterwards. Measurements were conducted in DTS1070 cells. The ζ‐potential at pH 7 was obtained by fitting the data points with a Boltzmann function (sigmoidal curve) in Origin 2024b with standard settings.

### Magnetic Biphenyl and PCB Extraction

Biphenyl and PCBs were dissolved in acetone at concentrations of 100 µm followed by dilution with DI water to obtain 1 µm solutions in DI water with 1 vol% of acetone (this ensures solubility, especially for the low‐soluble PCB 77).^[^ ^54^, ^55^ ^]^ These were titrated to pH 7 with aqueous 0.25 m NaOH and HCl solutions. Respectively, 7 mL of contaminant solution was given to 5.0 ± 0.1 mg (except when the mass was varied) of SPIONs, which were predispersed in 50 µL of acetone. These dispersions were put in a vortex mixer ZX4 by Velp Scientifica for 10 min at 500 rpm followed by 30 min of magnetic separation of the SPIONs carrying the contaminants. For the competitive extraction of biphenyl and the PCBs, equal volumes of 400 µm solutions in acetone were mixed. The resulting solution containing each contaminant at 100 µm was diluted accordingly with DI water to obtain an aqueous solution with 1 vol% of acetone containing each molecule at 1 µm. This solution was titrated to pH 7 and treated with SPIONs as described before. For the magnetic extraction from river water, the contaminants‐containing acetone solution was diluted with river water instead. Here, no titration was conducted to maintain the natural pH, which was measured at 7.95. For recycling the SPIONs were magnetically separated from the hexane solution that was later quantified, washed with fresh hexane, and dried in the oven at 80 °C. These particles were then wetted with 50 µL of acetone again and iteratively applied to contaminant‐spiked DI water. All magnetic extractions of individual biphenyl and PCBs as well as the competitive study containing all four contaminants were conducted in hexaplicates (split into multiple independent measurements). Comparison of DI to river water as well as the study of varying starting mass of SPIONs were done in triplicates, while recycling was done in quintuplicates, but one multiplicate was lost in the last step for characterization purposes.

### Quantification of Magnetically Extracted Biphenyl and PCBs

After magnetically collecting the SPIONs from water, they were transferred to 10 mL of hexane and sonicated for 10 min at 25 °C in a Sonocool 255 from Bandelin to wash off any biphenyl or PCB. Aliquots of 1.5 mL were centrifuged at 15000 rpm at 20 °C for 10 min in a Mikro 200R from Hettich to remove any SPIONs. 1.125 mL of the contaminant (i.e., analyte) hexane solution where mixed with 0.375 mL of PCB 209 (referred to as internal standard) à 4 µm in hexane to result in an analyte solution containing 1 µm of internal standard. This solution was investigated by GC‐MS. Based on method recommendations by the United States Environmental Protection Agency on quantification of PCBs,^[^ ^49^ ^]^ 2 µL were splitlessly injected at 270 °C via an AOC 20i autosampler into a GC‐MS‐QP2010 SE all from Shimadzu. Via helium as carrier gas at a total flow rate of 10 mL min^−1^ the analytes were separated by a Zebron ZB‐5Plus column from Phenomenex (30 m length, 0.25 mm diameter, and 0.25 µm coating thickness). The column oven was heated from 80 °C (held for 1 min) to 300 °C (held for 5 min) at a rate of 22 °C min^−1^. Finally, *m/z* ratios between 30 and 600 Da were recorded with a detector voltage of 0.99 ± 0.3 kV. The unknown analyte concentrations were determined by referring the respective peak areas in the specifically ion‐filtered chromatograms (quantifier *m/z* ratios: 154 Da (biphenyl), 188 Da (PCB 2), 222 Da (PCB 14) and 292 Da (PCB 77)) to the peak area of the internal standard PCB 209 (quantifier *m/z* ratios: 497 or 498 Da). Previously, internal calibration series of varying analyte (1.5, 1, 0.75, 0.5, 0.25, and 0.1 µm) and constant internal standard (1 µm) concentrations were established in triplicates (see Figure S4, Supporting Information). All analyses including linear regression were performed via GCMSsolution 4.44 from Shimadzu.

### Statistical Analysis

All data carrying uncertainty given in the manuscript and Supporting Information are displayed as mean ± standard deviation. Where appropriate, the uncertainty was propagated via Gaussian error propagation. The sample size of the removal performance data determined from GC‐MS measurements given in Figures 2 and 3 is *n* = 6, given in Figure 4 is *n* = 3, and given in Figure S6 (Supporting Information) is *n* = 5 (*n* = 4 in the last cycle). The ATR‐FTIR spectra in Figures S1 and S5 (Supporting Information) are given representatively and the thermogravimetric data as well as grafting densities in Figures S2 and S5 (Supporting Information) were determined from *n* = 3 (except the thermogravimetric analysis of the recycling, which is *n* = 1). In Figure S3 (Supporting Information), Z‐averages were determined from technical *n* = 3 as well as ζ‐potentials from technical *n* = 5 of one sample per pH value. GC‐MS chromatograms and calibrations in Figure S4 (Supporting Information) are representative, while the calibrations were always determined from *n* = 3. Data treatment is described in detail with the respective methods before.

### Ethical Statement

No ethics‐relevant experiments were performed in this work. Therefore, no ethical approval is required.

## Conflict of Interest

The authors declare no conflict of interest.

## Author Contributions

L.M. and M.H. performed conceptualization. L.M., A.Z., H.G., and D.K. performed the methodology. L.M., H.G., and E.H. performed formal analysis. L.M., A.Z., A.H., and J.V. performed the investigation. L.R., D.Z., A.H., and M.H. provided resources. L.M. Wrote the original draft. All the authors wrote, reviewed, and Edited the original draft. L.M. and E.H. performed visualization. D.Z., A. H., and M.H. performed supervision. L.M. and M.H. performed project administration. L.M. and M.H. provided funding acquisition.

## Supporting information



Supporting Information

## Data Availability

All source and measurement data supporting this study are available in the Figshare data repository (https://doi.org/10.6084/m9.figshare.26496859).^[^ ^56^ ^]^
