# Supplementary material for: Tailorable Nanoparticles for Magnetic Water Cleaning of Polychlorinated Biphenyls
Source: Small Methods. 2025 May 8;10(2):2500537. doi: 10.1002/smtd.202500537 (PMC12825353; doi:10.1002/smtd.202500537)
Supplement: Supplementary file 1 — Supporting Information [file SMTD-10-2500537-s001.docx]

Supporting Information

Tailorable Nanoparticles for Magnetic Water Cleaning of Polychlorinated Biphenyls

*Lukas Müller*, Anna Zehetmeier, Anna Höfling, Henrik Gaß, Johannes Voß, Daniel Krappmann, Linda Rockmann, Elias Harrer, Dirk Zahn, Andreas Hirsch, Marcus Halik**

Lukas Müller, Anna Zehetmeier, Anna Höfling, Henrik Gaß, Johannes Voß, Linda Rockmann, Marcus Halik

Organic Materials & Devices, Institute of Polymer Materials

Interdisciplinary Center for Nanostructured Films (IZNF)

Friedrich-Alexander-Universität Erlangen-Nürnberg

91058 Erlangen, Germany

*Correspondence: lukas.lm.mueller@fau.de, marcus.halik@fau.de

Daniel Krappmann, Andreas Hirsch

Chair of Organic Chemistry II

Friedrich-Alexander-Universität Erlangen-Nürnberg

91058 Erlangen, Germany

Elias Harrer, Dirk Zahn

Computer Chemistry Center, Chair of Theoretical Chemistry

Friedrich-Alexander-Universität Erlangen-Nürnberg

91052 Erlangen, Germany

**Calculation of electrostatic potential maps**

All calculations were carried out using density functional theory as implemented in the ORCA 5.0.4 quantum chemistry software package.^[1,2]^ Geometries were optimized using the PBE0 density functional^[3]^ in combination with the D4 dispersion correction.^[4]^ Furthermore, a def2-TZVP basis set was employed.^[5,6]^ Acceleration of the calculations was achieved by applying resolution of identity approximation^[7]^ and chain of sphere exchange.^[8]^ To confirm the optimization resulted in a minimum energy structure, the vibrational frequencies of all optimized geometries were calculated by applying the harmonic oscillator approximation. Zero imaginary frequencies indicated a minimum energy geometry. Electrostatic potential maps were obtained via the Multiwfn 3.8 software.^[9,10]^ All isosurface plots were generated by the VMD 1.9.3 software.^[11]^ To visualize the electrostatic potential, we use a molecular surface generated by imposing an isovalue of the electron density of 0.001 (in atomic units) – which corresponds to about half of the surface-nucleus distances of molecular surfaces generated from van-der-Waals radii.

**Characterization of SPION systems**

The morphology, maghemite crystal structure (γ-Fe_2_O_3_) as well as magnetic properties of the superparamagnetic iron oxide nanoparticles (SPIONs) were investigated previously via transmission electron microscopy, electron diffraction and vibrating sample magnetometer superconductive quantum interference device measurements.^[12,13]^ They have an average primary diameter of 10.7 nm assuming maghemite bulk density and spherical shape calculated from their Brunauer-Emmett-Teller (BET) specific surface area of 115.1 m^2^ g^-1^ measured via N_2_ gas adsorption.

As **Figure S1** shows via attenuated total reflectance Fourier transform infrared spectroscopy (ATR-FTIR), characteristic vibrational bands of the phosphonic acids (e.g., 2956, 2915, 2847 and 1467 cm^-1^ for CH_3_ and CH_2_ as well as convoluted peaks between 1185 and 880 cm^-1^ for PO_x_) are transferred to the nanoparticle surface indicating a successful self-assembled monolayer (SAM) formation. By referring the vibrational absorbance bands corresponding to asymmetrical CH_2_ (about 2925 cm^-1^) to CH_3_ vibrations (about 2955 cm^-1^) one can roughly estimate the ratio of PAC_3_Imi+ and PAC_16_, while the ratio of PAC_3_Imi+ and PAC_10_Ph can be approximated by dividing asymmetrical CH_2_ and aromatic CH (about 3020 cm^-1^) vibrational absorbance intensities. Thereby, a ligand share of on average more than 90 % of PAC_16_ and PAC_10_Ph is estimated in the binary SAM systems. The relatively smaller intensities as well as less convoluted PO_x_ vibrational bands for PAC_3_Imi+@SPIONs go in hand with the thermogravimetric analysis (**Figure S2** a) ), which reveals a smaller grafting density on the surface of 1.21 ± 0.08 nm^-2^ due to electrostatic repulsion (Figure S2 b) ). For the uncharged PAC_16_ and PAC_10_Ph densities of 2.25 ± 0.06 and 2.17 ± 0.11 nm^-2^ can be estimated, respectively. The less dense grafting of PAC_3_Imi+ on the surface is used in a second self-assembly step to be filled up and partially replaced in order to yield overall densities of 1.94 ± 0.32 nm^-2^ for PAC_3_Imi+/PAC_16_@SPIONs and 1.77 ± 0.08 nm^-2^ for PAC_3_Imi+/PAC_10_Ph@SPIONs. These values strongly depend on the available specific surface area on the SPIONs. Since the BET-determined specific surface area after dispersion in the functionalization solvent, methanol, is similar to before (114.8 m^2^ g^-1^), the original value is assumed to be available for the phosphonic acids. Overall, the thermogravimetric analyses reveal high thermal stability of the SAMs up to 200 °C indicating strong binding. In terms of hydrodynamic diameters in water all systems show Z-averages one magnitude larger than the primary diameter with even stronger agglomeration around the isoelectric points (see **Figure S3**). This is beneficial for efficient magnetic separation while still providing large surface area. Since this process is carried out from aqueous solutions titrated to pH 7, the ζ-potential at this pH of untreated SPIONs (-11.3 mV) can be either shifted up by PAC_3_Imi+ (+0.3 mV) or down by PAC_16_ (-36.3 mV) and PAC_10_Ph (-33.8 mV). The low ζ-potential of PAC_3_Imi+@SPIONs is likely due to free hydroxy groups on the surface as indicated by the low grafting density in combination with the positive charges of the imidazolium ions. The binary SAMs provide tuned values in between the extrema of -27.9 and -26.5 mV for PAC_3_Imi+/PAC_16_@SPIONs and PAC_3_Imi+/PAC_10_Ph@SPIONs, respectively.


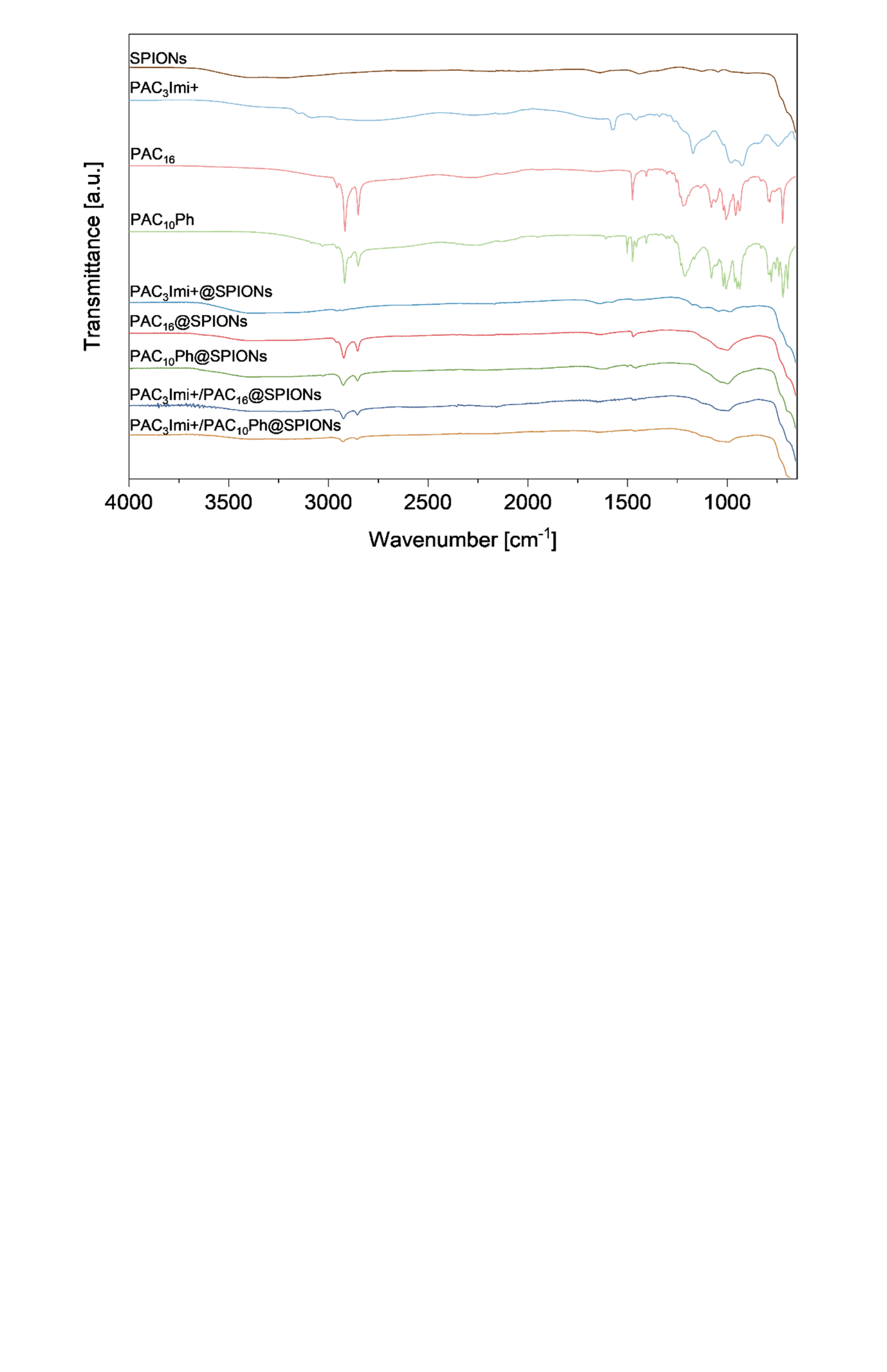


**Figure S1:** Representative ATR-FTIR spectra of reference SPIONs, reference phosphonic acids and functionalized SPIONs show successful attachment of the molecules to the surface of the nanoparticles. The characteristic vibrational bands (e.g., 2956, 2915, 2847 and 1467 cm^-1^ for CH_3_ and CH_2_ as well as convoluted peaks between 1185 and 880 cm^-1^ for PO_x_) are transferred to the SPIONs.


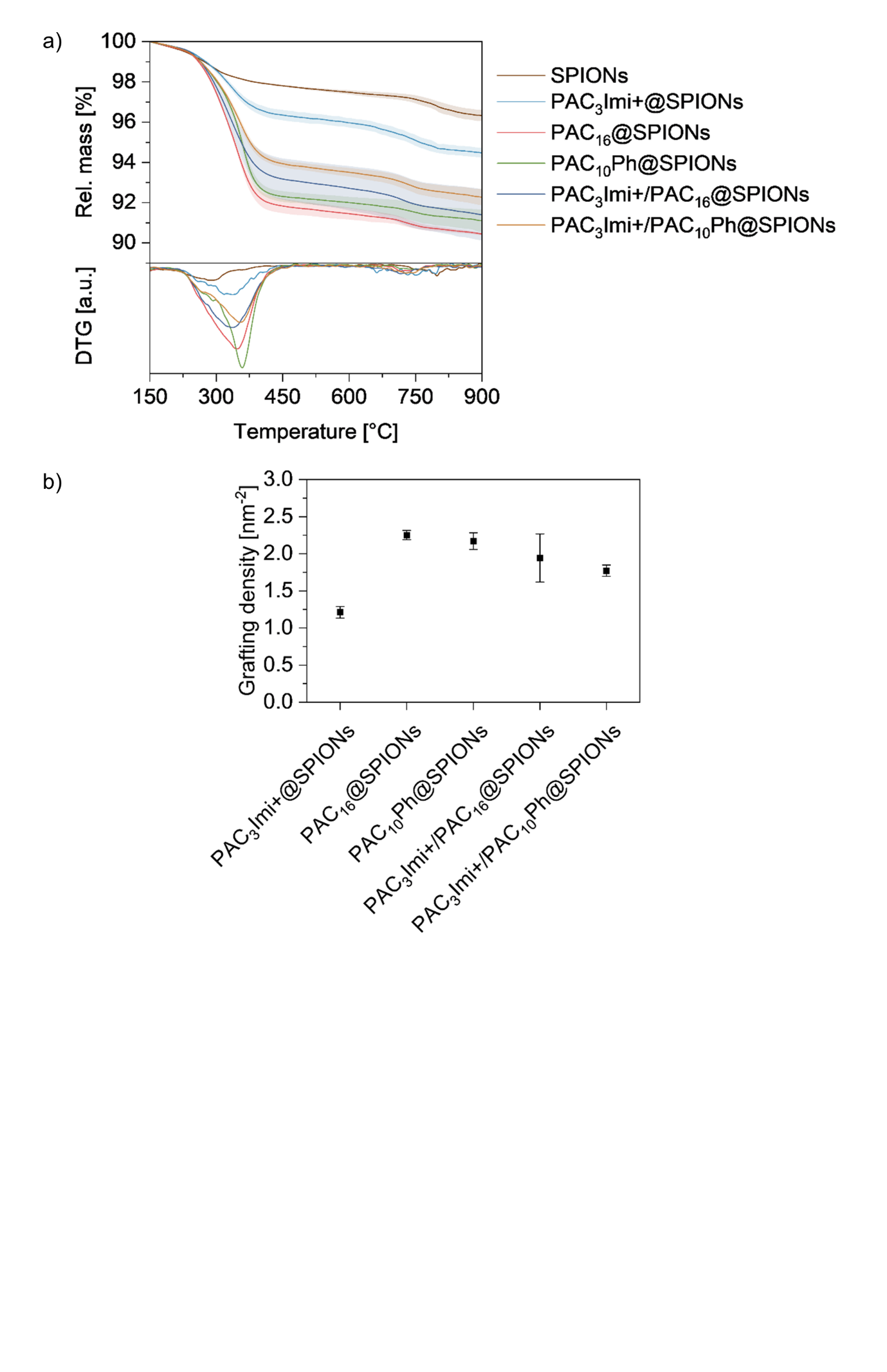


**Figure S2**: a) Thermogravimetric analyses and first derivatives (DTG) of reference and functionalized SPIONs enable b) estimation of surface grafting densities of the respective phosphonic acids and phosphonic acid mixtures on the nanoparticles. Due to the positive charges, PAC_3_Imi+ shows a much lower grafting density than PAC_16_ and PAC_10_Ph, which can be filled up by applying PAC_16_ and PAC_10_Ph in a second step to create the binary SAMs. Data are represented as mean ± standard deviation (n=3).


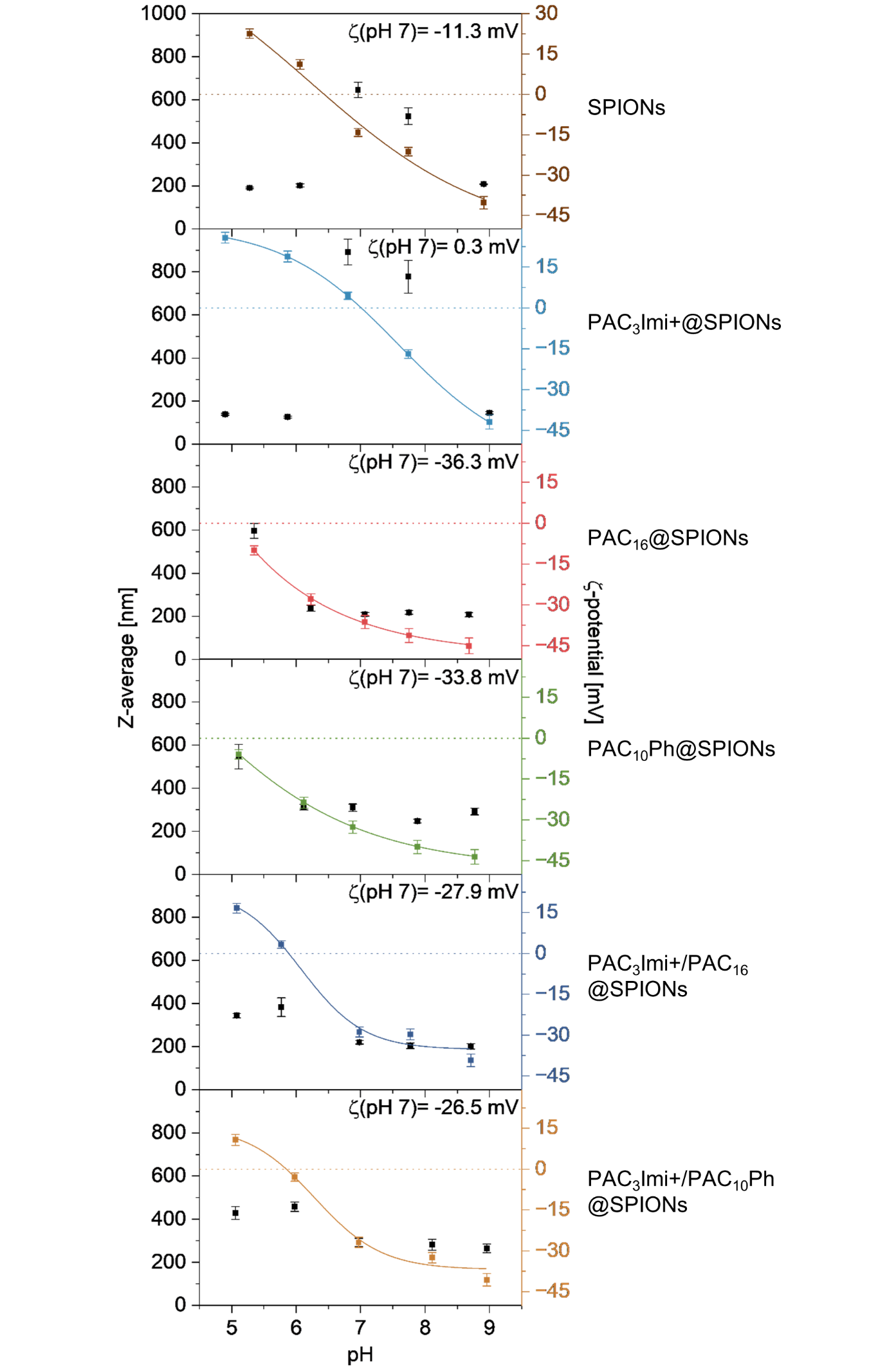


Figure S3: Hydrodynamic diameters and ζ-potentials of reference and functionalized SPIONs measured via dynamic and electrophoretic light scattering at various pH values (lines indicate fits of the ζ-potentials by a Boltzmann function to determine values at pH 7). While PAC_3_Imi+ shifts the isoelectric point of the particles to a higher pH value, PAC_16_ and PAC_10_Ph do the opposite. The binary systems show a tuned behavior in between the extrema. Data are represented as mean ± (Gaussian error propagated) standard deviation (technical n=3 for Z-averages and technical n=5 for ζ-potentials).

**GC-MS chromatograms and calibrations of biphenyl and the PCB analytes**


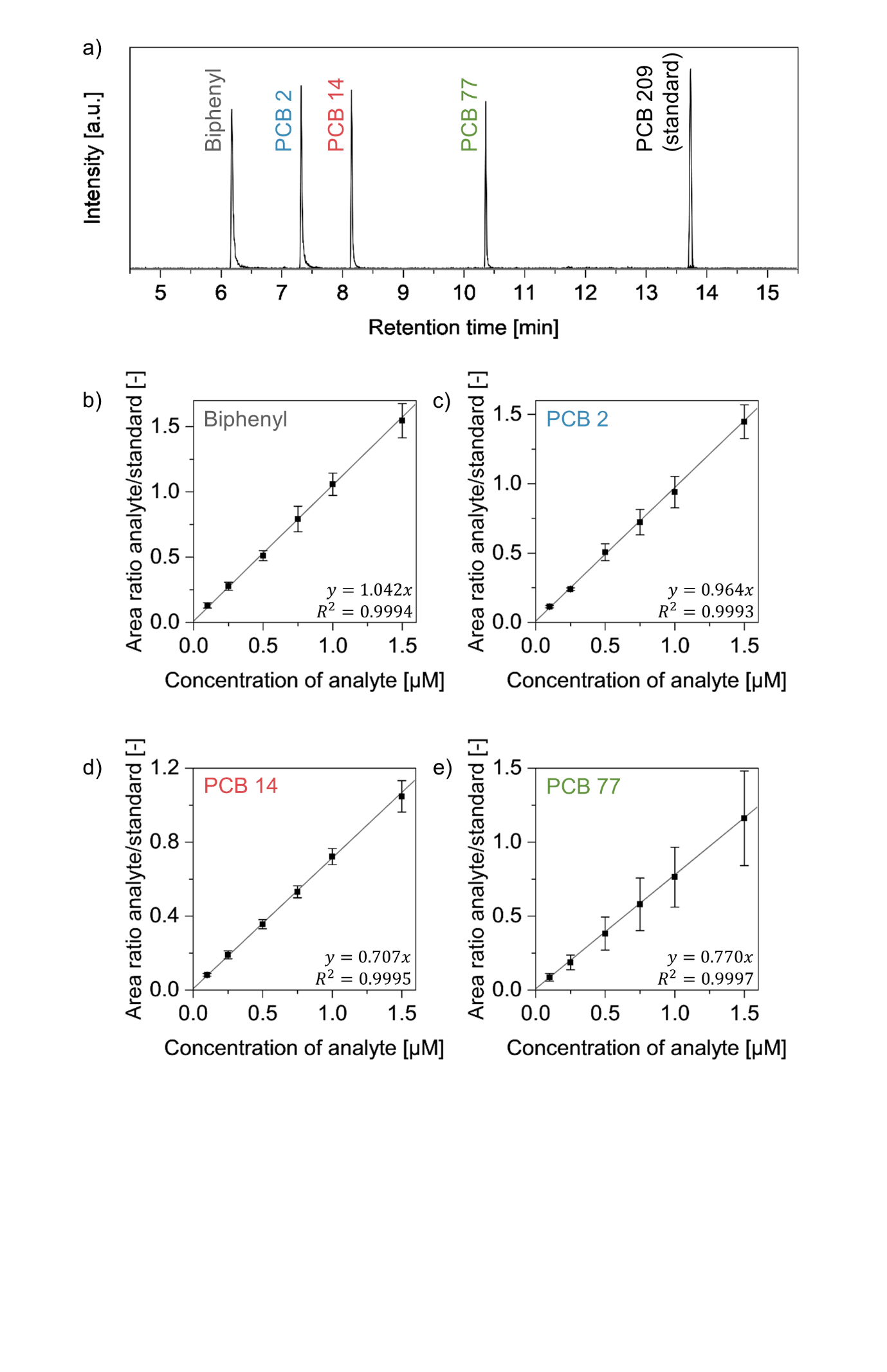


**Figure S4**: a) Representative quantifier ion-filtered chromatograms of biphenyl (*m/z* 154 Da), PCB 2 (*m/z* 188 Da), PCB 14 (*m/z* 222 Da), PCB 77 (*m/z* 292 Da) and internal standard PCB 209 (*m/z* 497 or 498 Da) each at 1 µm in hexane. b)-e) showing representative calibration series relating areas of the analytes in varying concentrations to the internal standard at a fixed concentration with linear fits. These were used for quantification of unknown concentrations later on. Data are represented as mean ± standard deviation (n=3).

**Recycling of PAC_3_Imi+/PAC_10_Ph@SPIONs**


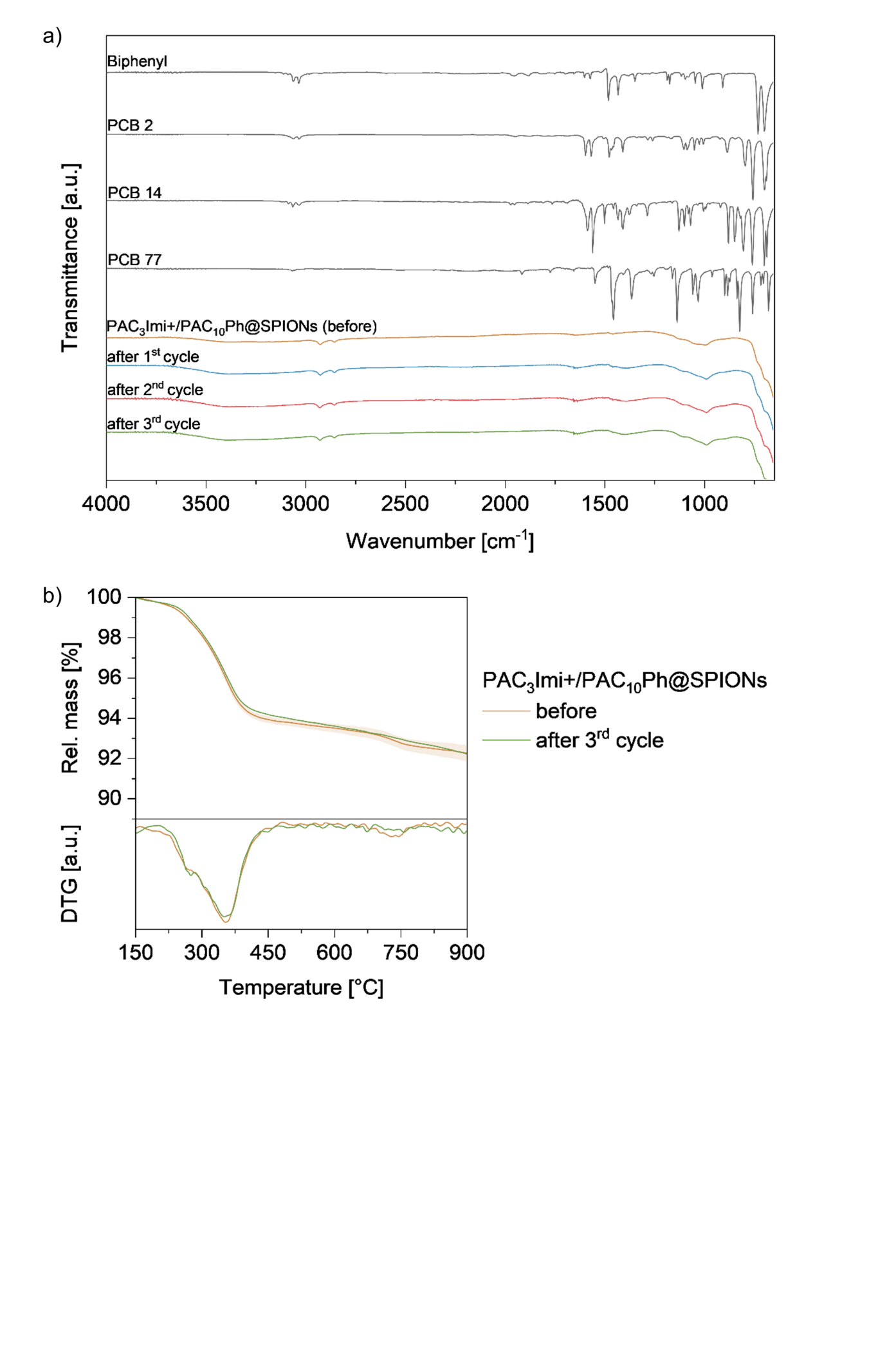


**Figure S5**: a) ATR-FTIR spectra and b) thermogravimetric analyses with first derivatives (DTG) of recycled PAC_3_Imi+/PAC_10_Ph@SPIONs show no indication for degradation nor remaining biphenyl or PCBs on the nanoparticle surface. Thermogravimetric data in b) before recycling is shown as mean ± standard deviation (n=3 before and n=1 after recycling).

**
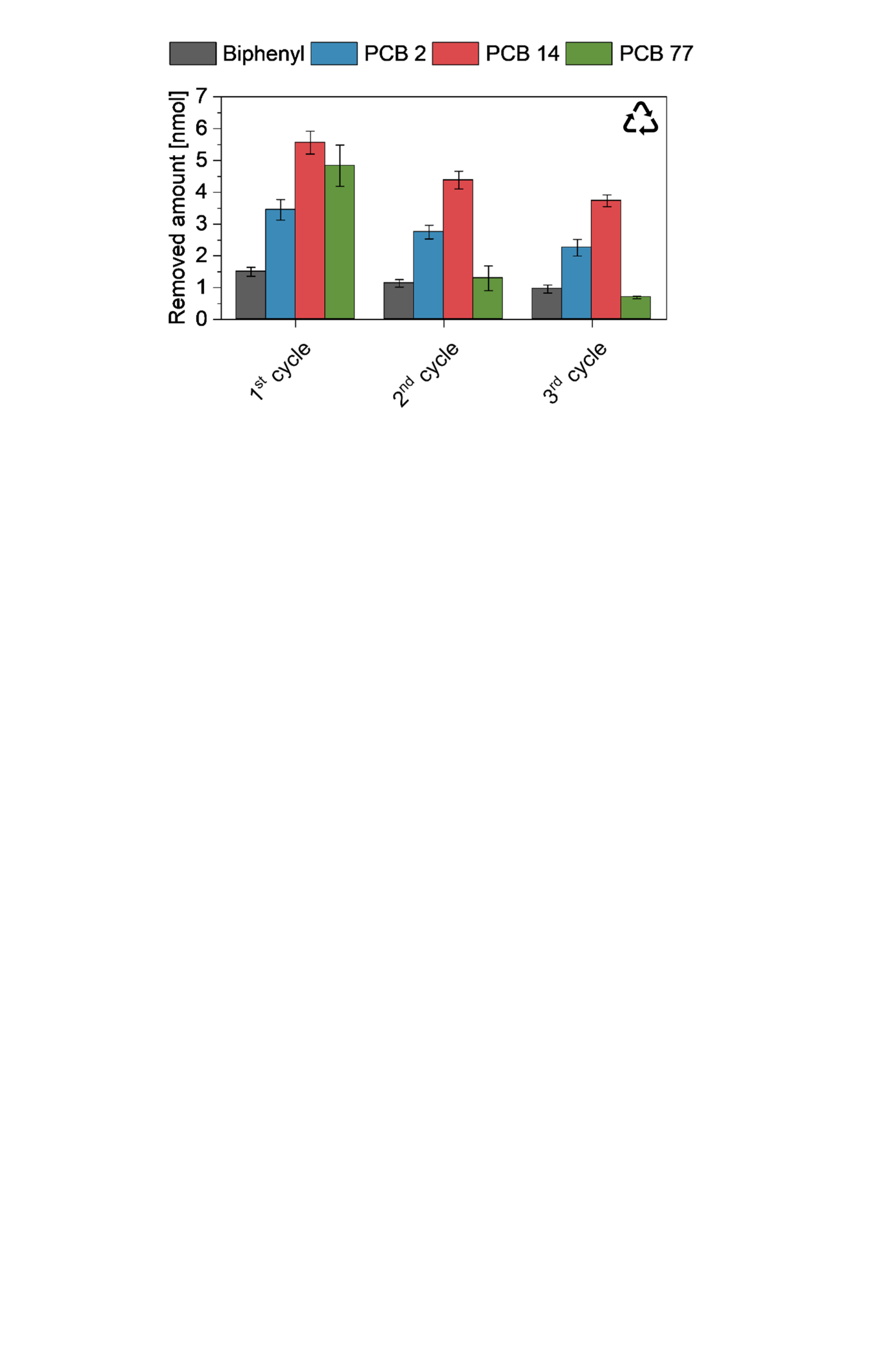
**

**Figure S6**: Magnetically removed amounts of biphenyl and PCBs from mixtures by PAC_3_Imi+/PAC_10_Ph@SPIONs after recycling. Aqueous solutions of 7 mL initially contained 7 nmol (1 µm) of each pollutant, i.e., 28 nmol overall, at neutral pH and were treated with recycled 5.0 ± 0.1 mg of the surrogate SPION system. The particles can be reused over multiple cycles without major losses in efficiency regarding three out of four pollutants. The strong decrease in removal of PCB 77 might be due to residual traces of hexane in the SAM. Data are represented as mean ± standard deviation (n=5 for 1^st^ and 2^nd^ cycle, n=4 for 3^rd^ cycle).

**References**

[1] F. Neese, F. Wennmohs, U. Becker, C. Riplinger, The ORCA quantum chemistry program package, *J. Chem. Phys.* **2020**, *152*, 224108.

[2] F. Neese, Software update: The ORCA program system—Version 5.0, *WIREs Comput. Mol. Sci.* **2022**, *12*, e1606.

[3] C. Adamo, M. Cossi, V. Barone, An accurate density functional method for the study of magnetic properties: The PBE0 model, *J. Mol. Struct. THEOCHEM* **1999**, *493*, 145.

[4] E. Caldeweyher, S. Ehlert, A. Hansen, H. Neugebauer, S. Spicher, C. Bannwarth, S. Grimme, A generally applicable atomic-charge dependent London dispersion correction, *J. Chem. Phys.* **2019**, *150*, 154122.

[5] F. Weigend, R. Ahlrichs, Balanced basis sets of split valence, triple zeta valence and quadruple zeta valence quality for H to Rn: Design and assessment of accuracy, *Phys. Chem. Chem. Phys.* **2005**, *7*, 3297.

[6] F. Weigend, Accurate Coulomb-fitting basis sets for H to Rn, *Phys. Chem. Chem. Phys.* **2006**, *8*, 1057.

[7] R. A. Kendall, H. A. Früchtl, The impact of the resolution of the identity approximate integral method on modern ab initio algorithm development, *Theor. Chem. Acc.* **1997**, *97*, 158.

[8] F. Neese, F. Wennmohs, A. Hansen, U. Becker, Efficient, approximate and parallel Hartree-Fock and hybrid DFT calculations. A “chain-of-spheres” algorithm for the Hartree-Fock exchange, *Chem. Phys.* **2009**, *356*, 98.

[9] T. Lu, F. Chen, Multiwfn: A multifunctional wavefunction analyzer, *J. Comput. Chem.* **2012**, *33*, 580.

[10] J. Zhang, T. Lu, Efficient evaluation of electrostatic potential with computerized optimized code, *Phys. Chem. Chem. Phys.* **2021**, *23*, 20323.

[11] W. Humphrey, A. Dalke, K. Schulten, VMD: Visual Molecular Dynamics, *J. Mol. Graph.* **1996**, *14*, 33.

[12] M. Sarcletti, H. Park, J. Wirth, S. Englisch, A. Eigen, D. Drobek, D. Vivod, B. Friedrich, R. Tietze, C. Alexiou, D. Zahn, B. Apeleo Zubiri, E. Spiecker, M. Halik, The remediation of nano-/microplastics from water, *Mater. Today* **2021**, *48*, 38.

[13] M. Sarcletti, Friedrich-Alexander-Universität Erlangen-Nürnberg, *Iron Oxide Nanoparticles – Their Functionalization and Their Use as Sorbent for Hydrophobic Contaminants*, **2021**.
